# Delivering the National Diabetes Prevention Program: Assessment of Outcomes in In-Person and Virtual Organizations

**DOI:** 10.1155/2023/8894593

**Published:** 2023-10-26

**Authors:** Elizabeth K. Ely, Boon Peng Ng, Michael J. Cannon

**Affiliations:** ^1^Division of Diabetes Translation, Centers for Disease Control and Prevention, Atlanta, Georgia, USA; ^2^College of Nursing and Disability, Aging, And Technology Cluster, University of Central Florida, Orlando, Florida, USA

## Abstract

The Centers for Disease Control and Prevention's Diabetes Prevention Recognition Program (DPRP) has helped organizations deliver the National Diabetes Prevention Program (National DPP) lifestyle change program for over 10 years. Four delivery modes are now approved: in person, online (self-paced, asynchronous delivery), distance learning (remote, synchronous delivery), and combination (hybrid delivery using more than one delivery mode). We assessed outcomes using data from 333,715 participants who started the 12-month program between January 1, 2012, and December 31, 2018. The average number of sessions attended was highest for in-person participants (15.0), followed by online (12.9), distance learning (12.2), and combination (10.7). The average number of weeks in the program was highest for in-person participants (28.1), followed by distance learning (20.1), online (18.7), and combination (18.6). The average difference between the first and last reported weekly physical activity minutes reflected an increase for in person (42.0), distance learning (27.1), and combination (15.0), but a decrease for online (-19.8). Among participants retained through session 6 or longer, average weekly physical activity minutes exceeded the program goal of 150 for all delivery modes. Average weight loss (percent of body weight) was greater for in person (4.4%) and distance learning (4.7%) than for online (2.6%) or combination (2.9%). Average participant weight loss increased gradually by session for all delivery modes; among participants who remained in the program for 22 sessions, average weight loss exceeded the program goal of 5% for all delivery modes. In summary, if participants stay in the program, most have positive program outcomes regardless of delivery mode; they have some outcome improvement even if they leave early; and their outcomes improve more the longer they stay. This highlights the benefits of better retention and increased enrollment in the National DPP lifestyle change programs, as well as enhancements to online delivery.

## 1. Introduction

In 2010, the U.S. Congress authorized the Centers for Disease Control and Prevention (CDC) to create and lead the National Diabetes Prevention Program (National DPP). The National DPP is a partnership of public and private organizations collaborating to build a nationwide delivery system for a lifestyle change program (LCP) proven to prevent or delay the onset of type 2 diabetes in adults at high risk or those with prediabetes. The LCP is an evidence-based behavioral change intervention delivered by trained lifestyle coaches using a 12-month curriculum. Program quality assurance is overseen by CDC's Diabetes Prevention Recognition Program (DPRP), now entering its eleventh year of approving recognition for organizations delivering the National DPP LCP [1]. The DPRP requires that organizations offer a minimum of 22 sessions over a 12-month period, with sessions covering CDC-approved curriculum modules. The DPRP maintains a database of participant data submitted by delivery organizations, which allows for the calculation of specific performance metrics, such as percent weight loss and average weekly physical activity minutes, thus providing information on organizations' effectiveness when delivering the National DPP LCP.

In 2012, the first organization was recognized by the DPRP for in-person curriculum delivery. Since then, the DPRP has implemented three sets of revisions to its standards and operating procedures (DPRP Standards) [2]. Although each version of the DPRP Standards has adjusted the requirements for achieving various levels of CDC recognition, the collection of participant body weight and weekly minutes of physical activity has been constant throughout all versions. In 2015, the DPRP Standards approved virtual delivery of the LCP as an alternative to in-person delivery, with options for online or other (no specifications were given at the time for these options other than “virtually or via one or more distance-learning modalities”). In 2018, the DPRP Standards further categorized other delivery into distance learning or combination. According to the DPRP Standards, online delivery is defined as participants logging into self-paced program sessions via a computer, tablet, or smartphone (i.e., asynchronous) with required live lifestyle coach interaction offered to each participant no less than once weekly during the first 6 months and once monthly during the second 6 months. Distance learning delivery is defined as the lifestyle coach providing live delivery (i.e., synchronous) of session content in one location and participants calling in or videoconferencing from another location. Combination delivery is defined as delivery that uses a combination of any of the previously defined delivery modes for each individual participant by trained lifestyle coaches. Because this definition implies that at least one component of combination delivery must be synchronous, we can assume that most lifestyle coach interactions in these organizations are synchronous.

The objective of this paper is to describe the outcomes of retention (number of program sessions attended, number of weeks spent in the program, and number of days spent in the program), weight loss achieved during the program, and average weekly physical activity minutes reported by participants who had the opportunity to complete the 12-month LCP, with a specific focus on delivery mode. Analyzing these outcomes can help us understand program performance, identify what is working well, and highlight opportunities for improvement.

## 2. Materials and Methods

### 2.1. Population

The National DPP LCP is intended for individuals who (1) are at least 18 years old; (2) have been determined to be at high risk for developing type 2 diabetes based on the results of a qualifying blood test, a clinical diagnosis of gestational diabetes mellitus during a previous pregnancy, or a positive screening on the American Diabetes Association/CDC Prediabetes Risk Test; and (3) have a body mass index (BMI) of ≥ 25 kg/m^2^ (≥ 23 kg/m^2^ if Asian or Asian American). In addition, participants cannot be pregnant at the time of enrollment, nor can they have a previous diagnosis of type 1 or type 2 diabetes [2]. For this study, we limited the analysis to participants who enrolled in the LCP from January 1, 2012, through December 31, 2018. This allowed participants the opportunity to attend any sessions held during the required 12-month program duration, but also before the onset of the COVID-19 public health emergency, which began in 2020. Occasionally, participants who do not meet the BMI criterion are enrolled; we excluded these participants from this analysis.

The number of participants included in the study population and the retention analyses was 333,715; 127,092 were associated with in-person delivery, 196,670 with online delivery, 2,672 with distance learning delivery, and 7,281 with combination delivery. For the analysis of weight and of physical activity, we chose to use only participants who had at least two sessions with recorded weights as well as at least one session with recorded weekly physical activity minutes. This subset included 286,112 participants, which was 85.7% of the total study population. Of these, 112,633 were associated with in-person delivery, 165,052 with online delivery, 2,385 with distance learning delivery, and 6,042 with combination delivery.

### 2.2. Variables

Participant-level demographic variables included sex, age, race, and ethnicity. For this analysis, we used only the male and female categories because the number of participants with sex “not reported” accounted for ~0.1% of the total. We placed age into one of three categories: 18–44, 45–64, or 65+ years. The DPRP requires that delivery organizations ask participants to identify their ethnicity as Hispanic or Latino or not Hispanic or Latino. Participants are also asked if they identify as one or more of the following races: American Indian or Alaska Native, Asian or Asian American, Black or African American, Native Hawaiian or other Pacific Islander, or White. For this analysis, we combined ethnicity and race responses into mutually exclusive groups, first categorizing by ethnicity and then further categorizing not Hispanic or Latino by race. Participants who identify as more than one race were categorized as multiracial, and those who did not report ethnicity or race were reported as such. We calculated each participant's initial BMI using the participant's height and the first reported body weight. We then categorized these as 25 (or 23 if Asian or Asian American)–29 kg/m^2^, or ≥ 30 kg/m^2^, indicating that the person had overweight or had obesity, respectively.

In this analysis, we used the following session-level information: the date of each session, body weight reported on the session date, and total number of physical activity minutes recorded for the week prior to the session date.

### 2.3. Data Analysis

We examined retention in the lifestyle change program by three different metrics: (1) the number of sessions attended, (2) the number of weeks a participant spent in the program, and (3) the number of days a participant spent in the program, as well as how many days it took participants to reach each session number. To increase comparability across organizations, our retention analyses presented the number of sessions attended through 22, which is the required minimum. We know from data submitted that there are organizations offering more than the minimum of 22, but this number varies. We also capped the number of weeks attended at 44 due to organizations' variability in duration of delivery during the last 2 months of the program. Participants who spent more than 44 weeks in the program were included in this number. When assessing retention, the analysis was not restricted to participants who attended all 22 sessions and 44 weeks. In analyses of weight loss, for each session, we calculated participant weight loss as the difference between the weight at that session and their first recorded weight and then divided by the starting weight to get the percent weight loss. In analyses of physical activity, we calculated average weekly physical activity minutes for each participant by summing minutes for all sessions where valid minutes were recorded and then dividing by the number of those sessions. We also report the initial amount of physical activity reported, as well as the final amount reported.

All analyses were descriptive (means and medians), stratified by delivery mode, and conducted using SAS Enterprise 7.1 (SAS Institute Inc., Cary, NC).

## 3. Results

### 3.1. Enrollment and Retention

**Figure 1:**
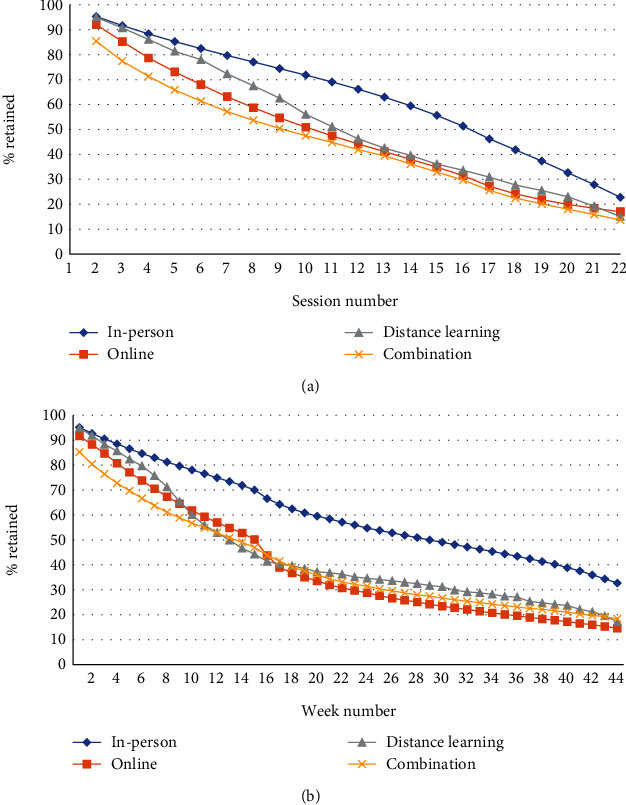
(a) Percent of participants in the National Diabetes Prevention Program (National DPP) lifestyle change program (LCP) retained at each session, by organization delivery mode. (b) Percent of participants in the National DPP LCP retained each week, by organization delivery mode.

**Figure 2:**
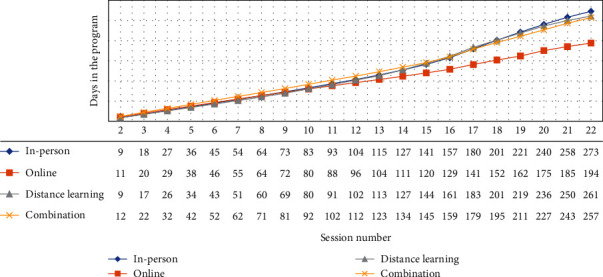
Average number of days to reach each session attended by participants in the National Diabetes Prevention Program lifestyle change program, by organization delivery mode.

Enrollment demographics for participants included in the retention analysis are listed in Table 1 and were consistent with what we previously described in detail [3]. This previous analysis showed that a higher proportion of men and participants aged 18-44 years enrolled in online and distance learning delivery programs than in in-person delivery programs. Retention measured by average number of sessions attended was highest for in-person participants (15.0), followed by online (12.9), distance learning (12.2), and combination (10.7) (Table 1). Of the four delivery modes, in-person delivery had the highest retention after attending one session (95.3%), while combination delivery had the lowest (85.4%) (Figure 1(a)). Retention through session 22 was highest among in-person participants (22.8%) and lowest among combination participants (13.7%) (Figure 1(a)). Retention measured by the average number of weeks in the program was highest for in-person participants (28.1), followed by distance learning (20.1), online (18.7), and combination (18.6) (Table 1). Retention through 44 weeks was highest among in-person participants (32.7%), followed by combination (18.4%), distance learning (17.2%), and online (14.6%) (Figure 1(b)). Participants enrolled in in-person programs were retained to 196.9 days, on average, which was higher than any other delivery mode (Table 1). Those enrolled in combination programs spent the least number of days in the program, on average, 130.5. When examining the average number of days it took participants to reach each session, we observed substantial differences by delivery mode (Figure 2, Table S2). Although the number of days was similar through approximately 8 sessions, online participants were reaching session 12 in 6–16 fewer days and were reaching session 22 in 63–79 fewer days, relative to other delivery modes.

### 3.2. Physical Activity

Table 2 shows the outcomes for the study population restricted to those who had at least two sessions with recorded weights and at least one session with recorded weekly physical activity minutes. The distribution of participants by demographic variables was similar to that of the larger study population (Table 1) and is found in Table S1.

First and last physical activity minutes recorded for participants were displayed regardless of which sessions were their first and last (Table 2). The first-session average weekly minutes was highest for online participants (183.0), followed by distance learning (170.3), combination (132.2), and in person (127.3). The last-session average weekly minutes were highest for distance learning participants (197.4), followed by in person (169.3), online (163.2), and combination (147.2). Only online participants had a lower last-session average of physical activity minutes than their first-session average, with a difference of -19.8 minutes. In-person participants showed the highest increase in first-to-last-session averages (42 minutes).

**Figure 3:**
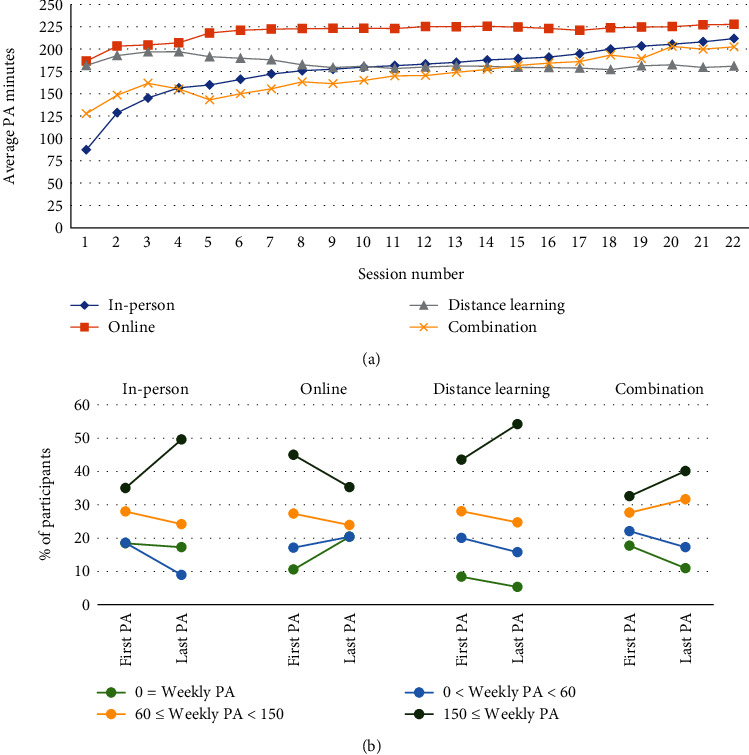
(a) Average weekly physical activity (PA) minutes for participants in the National Diabetes Prevention Program (National DPP) lifestyle change program (LCP), by session and organization delivery mode. The value for each session was calculated using PA minutes from participants still in the program at that session. (b) Proportion of participants in the National DPP LCP with first and last entry of weekly PA minutes in each specified interval, by organization delivery mode. Session numbers where the first and last recorded weekly PA minutes were reported varied for individual participants. Of note, the percentage change in those reaching the program goal of 150 minutes per week can be seen in the dark green line.

Average physical activity minutes increased with the number of sessions completed for all delivery modes except distance learning (Figure 3(a), Table S3). By session 6, participants in all delivery modes were averaging at least 150 minutes per week. Across all 22 sessions, average physical activity minutes were highest among participants enrolled in online organizations and peaked for these participants at session 22, with 228 reported minutes. The proportion of participants meeting the 150-minute goal increased from the first to last session for all delivery modes except online (Figure 3(b)).

### 3.3. Weight Loss

**Figure 4:**
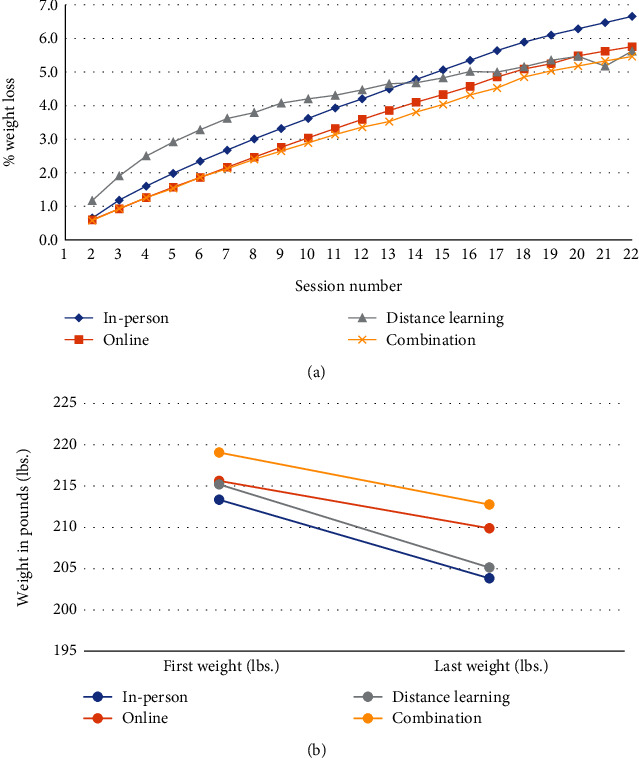
(a) Average weight loss per session for participants in the National Diabetes Prevention Program (National DPP) lifestyle change program (LCP), by organization delivery mode. (b) Average first and last recorded weight for participants in the National DPP LCP, by organization delivery mode. The value for each session was calculated using weights from participants still in the program at that session who reported a weight at that session.

For all delivery modes, the average participant percent weight loss gradually increased as the session number increased (Figure 4(a), Table S4). Average percent weight loss among those who attended through session 22 was highest among in-person participants (6.7%) and lowest among combination participants (5.5%), but participants in all delivery modes met the average percent weight loss program goal of 5%. On average, in-person participants met the 5% goal sooner (at session 15) than those in programs using other delivery modes. Participants in programs using combination delivery did not meet the 5% average goal until session 19 (Figure 4(a), Table S4), approximately 70 days later (Figure 2, Table S2).

**Figure 5:**
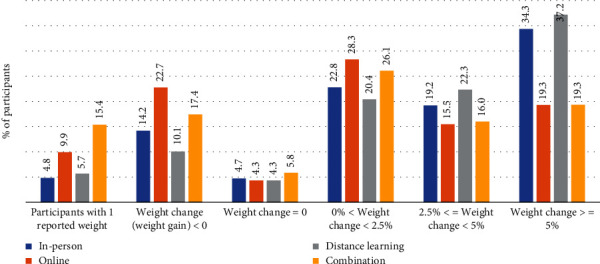
Weight loss outcomes for all participants in the National Diabetes Prevention Program lifestyle change program, by organization delivery mode.

When comparing average initial weight to average final weight, we saw a decrease across all delivery modes (Figure 4(b), Table S5). The decrease in average weight was greatest for distance learning participants (10.1 lbs.), followed by in person (9.5 lbs.), combination (6.3 lbs.), and online (5.8 lbs.) (Table 2). Overall, participants enrolled in distance learning and in-person organizations represented the highest percentage of those meeting the 5% weight loss goal, 37.2% and 34.3%, respectively (Figure 5). Those enrolled in online and combination organizations represented the highest percentage of participants who gained weight during the lifestyle change program, 22.7% and 17.4%, respectively. Organizations using combination delivery had the highest percentage of participants with only one recorded weight (15.4%), while in-person organizations had the lowest percentage of participants with only one recorded weight (4.8%).

**Figure 6:**
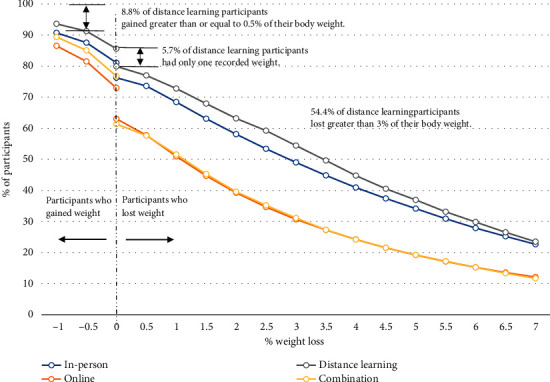
Cumulative distribution of weight loss outcomes for all participants in the National Diabetes Prevention Program lifestyle change program, by organization delivery mode.

Figure 6 displays cumulative weight loss by delivery mode for all participants. Distance learning organizations had the smallest percentage of participants who lost ≤ 0% of their initial body weight (i.e., gained weight or lost no weight) (14.4%), whereas online organizations had the highest (27.0%). In addition, distance learning organizations had the highest percentage of participants who achieved more than 7% weight loss (23.5%). With respect to meeting the program goal of losing at least 5% of one's body weight, distance learning participants achieved this at the highest rate (36.9%), followed by in person (34.2%), and combination and online (19.2%). Examples are provided to help with interpretation. Results are cumulative (e.g., participants who lost 6% of their weight would also be included in the percent of participants who lost 5% of their weight). The lines do not go to 100% because weight gain was truncated at 1% (i.e., weight loss of -1%).

## 4. Discussion

Enrolling and retaining participants in a voluntary, 12-month behavioral health intervention is inherently challenging. We found that the median time spent in the National DPP LCP was 6.6 months for in-person participants and a range of 2.9 to 3.5 months for participants using other delivery modes. Participants often face challenging personal, family, and work constraints that affect retention [4–6], but retention is also likely to be influenced by program factors such as the curriculum used, coach skills, program location, and class schedules. A recent survey of in-person participants found that, by far, the biggest factor associated with dropping out was class timing and scheduling [6], suggesting that flexibility of class offerings could be a key way to improve retention. Increased focus on individual participant goals has also been cited as something that might help retain people longer [7]. Emotional support and accountability are important to some participants as they move through the program [8]. In particular, organizations delivering behavior change interventions using digital platforms such as the Internet or mobile phones may find that lack of personal contact may be reason enough for some participants to drop out [8]. Technology-only (asynchronous) driven interventions may allow for easier participant disengagement [9]. Retention may be further improved by obtaining more feedback from participants, coaches, and current and prospective delivery organizations to identify and address other relevant factors.

Even though most participants dropped out before attending 22 sessions, results showed that, on average, participants in every delivery mode increased their physical activity (Figure 3(a), Table S3) and lost weight (Figure 4(a), Table S4) as they moved through the program. Despite average session attendance ranging from only 10.7 to 15.0 across the four delivery modes (Table 1), the percentage of participants losing some amount of weight was 76.3% (in person), 63.1% (online), 79.9% (distance learning), and 61.4% (combination) (Figures 5 and 6). Other translation studies have also found that participants lose weight incrementally the more time they spend in a program [10]. Furthermore, simply avoiding weight gain is highly protective against diabetes [11]. In addition, physical activity interventions have been shown to be independently associated with lowering diabetes risk [12], which contributed to DPRP's addition, in 2021, of an optional outcome measure combining physical activity with a lower weight loss threshold for the National DPP LCP [2]. Taken together, these findings suggest that participants will likely derive some health benefits even if they leave the program early, but that the benefits will increase the longer participants stay.

Across all delivery modes, we found that average weight loss reached ≥ 5% by session 19 (~5–7 months) (Figures 2 and 4(a)), and most participants reached ≥ 150 weekly physical activity minutes by approximately session 6 (Figure 3(a), Table S3). However, it is likely that greater time in the program enhances habit formation and longer-term behavioral change. The community guide recommended that programs have a minimum 3-month duration. The guide also concluded that higher intensity programs (e.g., more sessions) lead to greater reduction in new-onset type 2 diabetes, and that programs with longer core phase durations may be more effective [13]. In our study, as well as a previous study using DPRP data [14], greater time in the program led to greater average weight loss (with the exception of distance learning) and greater average physical activity minutes (Figures 4(a) and 3(a)). However, our data suggest that, in a real-world setting, retaining all participants to 12 months may not be feasible.

We found that in-person programs outperformed other delivery modes with respect to retention (Figures 1(a) and 1(b)), increases in physical activity (Table 2, Figure 3(b)), and (with the exception of distance learning) weight loss (Tables 2 and S5, Figure 4(b)). Distance learning participants, on average, showed the largest decrease from initial to final weight, 10.1 lbs. and 4.7% (Table 2). In-person and distance learning programs had the highest percent of participants achieving the 5% weight loss goal, 34.3% and 37.2%, respectively (Figure 5). We also noted that in-person participants met the average 5% weight loss goal at an earlier session (15) than other participants, as well as at an earlier time in the program (141 days, or about 4.6 months). Future analyses might examine if participants who achieve weight loss goals early go on to lose additional weight by the end of the program. Factors commonly associated with synchronous delivery (i.e., in-person and distance learning) such as the personal nature of the coach and peer interaction, as well as the sense of community, might contribute to better outcomes. A review of weight loss interventions that use a personal contact component, in addition to mobile phone technology for delivery, was shown to be more effective than those that lacked personal interaction [15]. It should be noted that when online delivery of the lifestyle change program includes intensive coach support (other than live delivery), or support through participant groups or online social networks, results that align with the National DPP's programmatic weight loss goal of 5% can be achieved [16]. In our study, there may be differences in outcomes due to participant motivation, readiness to attend, and contextual barriers to full engagement. Our results suggest that these are all factors that could improve retention, as well.

Although in-person participants tended to have better program results overall, and outcomes associated with online participants need improvement, online enrollment has surpassed in-person enrollment [3]. The expansive reach of online programs can help scale the National DPP LCP, especially as we see that participants who stay in these programs lose weight and increase their physical activity. Furthermore, online and other virtual delivery modes have the potential to reduce barriers to program participation that are more often encountered by people who experience adverse social determinants of health—for example, barriers related to childcare, transportation, distance to the program, and discretionary time. Of course, access to technology can also be a barrier, so serious attempts to reduce health disparities through the National DPP will likely explore how to leverage technology to reach underserved populations.

There are several limitations associated with DPRP data. First, the DPRP collects data on only a limited number of variables to minimize the data collection burden on program delivery organizations. Second, certain data points, such as physical activity minutes, are self-reported by participants. The DPRP does not collect information as to how these minutes are measured or reported. In addition, we do not collect information on the specific methods used by virtual organizations to obtain participant weights. Third, the DPRP receives only what organizations report, although it performs quality control checks and audits when necessary. Fourth, the precise nature of combination delivery is often unknown to the DPRP and likely varies by organization (e.g., the percent of sessions attributed to one delivery mode vs. another), precluding strong conclusions about the results for this delivery mode.

Of note, we found the distance learning data challenging to interpret because, even though retention was lower than for in-person delivery and participants' average physical activity minutes per session did not increase over time, overall weight loss was slightly higher than that of in-person participants. This appears to be driven by a high degree of early weight loss, which subsequently drops off over time (Figure 4(a), Table S4). Our findings about distance learning delivery should be considered preliminary because the sample size is only 1%–2% of the in-person or online populations. In future analyses, we plan to examine distance learning in more depth, using data collected from the expanded number of organizations that delivered via this modality during the COVID-19 public health emergency.

Future analysis should focus on outcomes by delivery mode and participant demographics (sex, age, race/ethnicity). Given that certain populations (e.g., men and participants aged 18-44 years) enroll in virtual programs at a higher rate than in-person programs [3], it will be important to examine how this impacts overall delivery mode outcomes.

## 5. Conclusions

Since it began in 2012, the Diabetes Prevention Recognition Program has recognized over 4,000 organizations delivering the National DPP lifestyle change program to nearly 700,000 participants, via four different delivery modes. While scaling the program can help reach more of the 96 million Americans with prediabetes [17], we can also focus on participant retention and outcomes. Retention is inherently challenging, but various program changes, such as the curriculum, coach skills, class schedules, and even a change in class location, can improve retention. Participants in in-person programs had the best retention, and participants in in-person and distance learning programs had the highest weight loss, suggesting that the essential features of synchronous delivery might be applied to other delivery modes. Nevertheless, if participants stay in the program long enough, most have positive program outcomes regardless of delivery mode. Importantly, most participants lose weight and increase physical activity if they leave the program early, but their outcomes improve more the longer they stay. These findings highlight the benefits of increased enrollment as well as better retention within the National DPP lifestyle change program.

## Figures and Tables

**Table 1:** Characteristics of participants who enrolled in the National Diabetes Prevention Program lifestyle change program by December 2018, by organization delivery mode.

	In-person		Online		Distance learning		Combination	
Population	127,092		196,670		2,672		7,281	
	*N*	%	*N*	%	*N*	%	*N*	%
Sex^a^								
Men	24,854	19.6	53,427	27.2	563	21.1	1,539	21.1
Women	102,060	80.4	143,083	72.8	2,109	78.9	5,739	78.9
Age group (years)								
18–44	23,891	18.8	84,404	42.9	756	28.3	1,564	21.5
45–64	67,683	53.3	103,977	52.9	1,601	59.9	3,839	52.7
65+	35,518	28.0	8,289	4.2	315	11.8	1,878	25.8
Race/ethnicity								
Hispanic/Latino	15,241	12.0	20,780	10.6	275	10.3	2,414	33.2
Non-Hispanic/Latino	91,857	72.3	166,438	84.6	2,337	87.5	4,365	59.9
American Indian/Alaska Native	1,604	1.3	1,133	0.6	8	0.3	28	0.4
Asian/Asian American	1,711	1.4	7,016	3.6	82	3.1	319	4.4
Black/African American	16,854	13.3	21,170	10.8	350	13.1	1,037	14.2
Native Hawaiian/other Pacific Islander	981	0.8	1,424	0.7	42	1.6	33	0.5
White	62,341	49.1	131,646	66.9	1,734	64.9	2,831	38.9
Multiracial	1,169	0.9	647	0.3	30	1.1	24	0.3
Race not reported	7,197	5.7	3,402	1.7	91	3.4	93	1.3
Hispanic/Latino not reported	19,994	15.7	9,452	4.8	60	2.3	502	6.9
Baseline body mass index (BMI)								
23–29 kg/m^2b^	27,612	21.7	47,158	24.0	610	22.8	1,113	15.3
≥30 kg/m^2^	99,480	78.3	149,512	76.0	2,062	77.2	6,168	84.7
	Mean (SD)	Median (IQR)	Mean (SD)	Median (IQR)	Mean (SD)	Median (IQR)	Mean (SD)	Median (IQR)
Retention								
Number of sessions	15.0 (8.3)	16.0 (8.0–21.0)	12.9 (11.4)	10.0 (4.0–17.0)	12.2 (7.6)	11.0 (6.0–19.0)	10.7 (8.8)	9.0 (3.0–17.0)
Number of weeks	28.1 (18.3)	28.9 (11.6–46.1)	18.7 (17.6)	15.0 (5.0–28.0)	20.1 (17.2)	12.4 (7.0–37.0)	18.6 (18.0)	13.0 (3.0–32.3)
Number of days	196.9 (128.2)	202.0 (81.0–323.0)	131.3 (122.9)	105.0 (35.0–196.0)	140.7 (120.5)	86.5 (49.0–259.0)	130.5 (126)	91.0 (21.0–226.0)

^a^Sex was not reported for 341 (~0.1%) participants. ^b^23–29 for Asian/Asian American participants; 25–29 for non-Asian/non-Asian American participants.

**Table 2:** Characteristics of participants who enrolled in the National Diabetes Prevention Program lifestyle change program by December 2018 and who had at least two sessions with recorded weights and at least one session with recorded weekly physical activity minutes, by organization delivery mode.

	In-person		Online		Distance learning		Combination	
Sample population	112,633		165,052		2,385		6,042	
Physical activity (PA) (minutes/week)	Mean (SD)	Median (IQR)	Mean (SD)	Median (IQR)	Mean (SD)	Median (IQR)	Mean (SD)	Median (IQR)
First reported PA	127.3 (150.3)	90 (20–180)	183.0 (198.0)	131 (50–240)	170.3 (168.7)	120 (40–250)	132.2 (188.6)	80 (10–165)
Last reported PA	169.3 (184.9)	140 (45–210)	163.2 (218.9)	90 (15–209)	197.4 (170.7)	150 (60–275)	147.2 (165.5)	100 (40–180)
Difference between first and last reported PA	42.0 (199.0)	10 (-35–115)	-19.8 (203.7)	-12 (-100–35)	27.1 (183.4)	0 (-47–100)	15.0 (191.3)	10 (-30–80)
Physical activity (minutes/week)	N	%	N	%	N	%	N	%
First reported PA								
Weekly PA = 0	20,760	18.4	17,434	10.6	200	8.4	1,070	17.7
0 < weekly PA < 60	20,911	18.6	28,261	17.1	478	20.0	1,334	22.1
60 ≤ weekly PA < 150	31,505	28.0	45,120	27.3	669	28.1	1,670	27.6
150 ≤ weekly PA	39,457	35.0	74,237	45.0	1,038	43.5	1,968	32.6
Last reported PA								
Weekly PA = 0	19,452	17.3	33,672	20.4	127	5.3	662	11.0
0 < weekly PA < 60	10,079	9.0	33,670	20.4	376	15.8	1,043	17.3
60 ≤ weekly PA < 150	27,238	24.2	39,471	23.9	589	24.7	1,913	31.7
150 ≤ weekly PA	55,864	49.6	58,239	35.3	1,293	54.2	2,424	40.1
Weight	Mean (SD)	Median (IQR)	Mean (SD)	Median (IQR)	Mean (SD)	Median (IQR)	Mean (SD)	Median (IQR)
First reported weight (lbs.)	213.3 (48.8)	205 (178–240)	215.6 (47.5)	208.0 (181.0–242.0)	215.2 (48.8)	206.0 (180.0–240.0)	219.1 (51.5)	211.0 (182.0–246.0)
Last reported weight (lbs.)	203.8 (48.0)	196 (170–230)	209.9 (47.3)	202.4 (175.9–236.0)	205.1 (47.7)	196.0 (172.0–230.0)	212.8 (51.0)	205.0 (176.0–239.0)
Difference between first and last reported weight (lbs.)	9.5 (12.2)	7.0 (2.0–15.0)	5.8 (10.8)	3.1 (0.0–9.3)	10.1 (11.1)	8.0 (2.8–15.0)	6.3 (10.9)	4.0 (0.0–10.0)
Percent weight lost between first and last weights (%)	4.4 (5.4)	3.5 (0.9–7.0)	2.6 (4.8)	1.6 (0.0–4.5)	4.7 (4.9)	3.9 (1.3–7.1)	2.9 (4.7)	1.8 (0.0–4.7)
Proportions with various percentages of weight loss	N	%	N	%	N	%	N	%
0% ≥ weight loss (includes weight gain)	20,880	18.5	48,219	29.2	354	14.8	1,647	27.3
0% < weight loss < 2.5%	25,458	22.6	51,329	31.1	498	20.9	1,843	30.5
2.5% ≤ weight loss < 5.0%	23,299	20.7	28,897	17.5	566	23.7	1,148	19.0
Weight loss ≥ 5.0%	42,996	38.2	36,607	22.2	967	40.6	1,404	23.2

## Data Availability

Data were collected under CDC's DPRP (OMB No. 0920-0909), for the primary purpose of evaluating the performance of organizations offering the National DPP lifestyle change program. Data are shared in aggregate form to inform technical assistance and enhance overall program outcomes.

## Supplementary Materials

Supplementary Materials Supplementary materials include the characteristics of participants who had at least two sessions with recorded weights and at least one session with recorded weekly physical activity minutes, by organization delivery mode (Table S1), average number of days to reach each session with 95% confidence limits, by delivery mode (Table S2), average weight loss and weekly physical activity minutes performed with 95% confidence limits, by session and delivery mode (Tables S3 and S4), and average first and last recorded weights, with 95% confidence limits, by delivery mode (Table S5).
