# Supplementary material for: Delivering the National Diabetes Prevention Program: Assessment of Outcomes in In-Person and Virtual Organizations
Source: J Diabetes Res. 2023 Oct 26;2023:8894593. doi: 10.1155/2023/8894593 (PMC10622599; doi:10.1155/2023/8894593)
Supplement: Supplementary Materials — include the characteristics of participants who had at least two sessions with recorded weights and at least one session with recorded weekly physical activity minutes, by organization delivery mode (Table S1), average number of days to reach each session with 95% confidence limits, by delivery mode (Table S2), average weight loss and weekly physical activity minutes performed with 95% confidence limits, by session and delivery mode (Tables S3 and S4), and average first and last recorded weights, with 95% confidence limits, by delivery mode (Table S5). [file 8894593.f1.zip › Table S4.docx]

Table S4. Average weight loss per session (with 95% confidence limits) for participants in the National Diabetes Prevention Program (National DPP) lifestyle change program, by organization delivery mode.

|  | **In-person** | | | **Online** | | | **Distance Learning** | | | **Combination** | | |
| --- | --- | --- | --- | --- | --- | --- | --- | --- | --- | --- | --- | --- |
| Session | Mean | Lower 95% | Upper 95% | Mean | Lower 95% | Upper 95% | Mean | Lower 95% | Upper 95% | Mean | Lower 95% | Upper 95% |
| 2 | 0.65 | 0.64 | 0.66 | 0.60 | 0.59 | 0.60 | 1.17 | 1.09 | 1.25 | 0.58 | 0.53 | 0.63 |
| 3 | 1.18 | 1.18 | 1.19 | 0.93 | 0.92 | 0.93 | 1.90 | 1.79 | 2.02 | 0.93 | 0.88 | 0.98 |
| 4 | 1.60 | 1.59 | 1.61 | 1.26 | 1.25 | 1.27 | 2.50 | 2.36 | 2.63 | 1.26 | 1.20 | 1.32 |
| 5 | 1.98 | 1.97 | 2.00 | 1.57 | 1.56 | 1.58 | 2.91 | 2.76 | 3.07 | 1.53 | 1.46 | 1.61 |
| 6 | 2.34 | 2.33 | 2.36 | 1.86 | 1.85 | 1.87 | 3.28 | 3.10 | 3.46 | 1.87 | 1.79 | 1.94 |
| 7 | 2.67 | 2.66 | 2.69 | 2.16 | 2.15 | 2.18 | 3.62 | 3.46 | 3.77 | 2.12 | 2.03 | 2.21 |
| 8 | 3.01 | 2.99 | 3.02 | 2.46 | 2.44 | 2.48 | 3.79 | 3.58 | 4.00 | 2.40 | 2.30 | 2.50 |
| 9 | 3.32 | 3.30 | 3.34 | 2.76 | 2.74 | 2.78 | 4.07 | 3.89 | 4.26 | 2.65 | 2.53 | 2.76 |
| 10 | 3.62 | 3.60 | 3.64 | 3.04 | 3.01 | 3.06 | 4.20 | 3.97 | 4.43 | 2.89 | 2.77 | 3.01 |
| 11 | 3.93 | 3.90 | 3.95 | 3.32 | 3.29 | 3.34 | 4.31 | 4.09 | 4.52 | 3.14 | 3.01 | 3.27 |
| 12 | 4.20 | 4.18 | 4.23 | 3.59 | 3.56 | 3.62 | 4.47 | 4.23 | 4.71 | 3.36 | 3.22 | 3.50 |
| 13 | 4.50 | 4.47 | 4.52 | 3.85 | 3.82 | 3.88 | 4.65 | 4.40 | 4.90 | 3.53 | 3.38 | 3.67 |
| 14 | 4.78 | 4.75 | 4.81 | 4.10 | 4.07 | 4.14 | 4.68 | 4.41 | 4.95 | 3.81 | 3.64 | 3.97 |
| 15 | 5.06 | 5.03 | 5.10 | 4.33 | 4.29 | 4.37 | 4.82 | 4.54 | 5.11 | 4.03 | 3.86 | 4.21 |
| 16 | 5.35 | 5.31 | 5.38 | 4.57 | 4.53 | 4.61 | 5.02 | 4.71 | 5.32 | 4.32 | 4.13 | 4.51 |
| 17 | 5.64 | 5.60 | 5.68 | 4.85 | 4.81 | 4.90 | 5.00 | 4.64 | 5.36 | 4.52 | 4.30 | 4.74 |
| 18 | 5.89 | 5.85 | 5.94 | 5.09 | 5.04 | 5.14 | 5.16 | 4.78 | 5.53 | 4.85 | 4.61 | 5.09 |
| 19 | 6.10 | 6.05 | 6.15 | 5.25 | 5.19 | 5.30 | 5.35 | 4.94 | 5.76 | 5.04 | 4.77 | 5.30 |
| 20 | 6.29 | 6.23 | 6.34 | 5.48 | 5.42 | 5.54 | 5.47 | 5.02 | 5.92 | 5.18 | 4.89 | 5.47 |
| 21 | 6.47 | 6.41 | 6.53 | 5.62 | 5.56 | 5.69 | 5.17 | 4.72 | 5.63 | 5.33 | 5.01 | 5.64 |
| 22 | 6.66 | 6.59 | 6.73 | 5.75 | 5.68 | 5.82 | 5.62 | 5.09 | 6.16 | 5.46 | 5.11 | 5.81 |
